# A classification of the aims of vaccination and its relevance to transgenerational justice

**DOI:** 10.7189/jogh.10.010341

**Published:** 2020-06

**Authors:** Lea Atzinger, Wolfram Henn

**Affiliations:** 1 Institute of Human Genetics, Saarland University, Homburg/Saar, Germany; 2 German Ethics Council (Deutscher Ethikrat), Berlin, Germany

Vaccines are well-established to be one of the most effective and cost-efficient proactive instruments of public health. The World Health Organization (WHO) estimates that measles vaccination programs alone have prevented over 21.1 million deaths globally between 2000 and 2017. Nevertheless, measles are still among the leading causes of childhood mortality worldwide [1]. However, especially in industrialized countries, it seems as though vaccines are about to become a victim of their own success where regionally successful vaccination programs have rendered the respective disease virtually unknown to the protected group [2]. As the general public typically does not witness the devastating manifestations of vaccine-preventable infectious diseases anymore, the consequences of contracting such a disease are often underestimated, and adherence to vaccination programs tends to diminish. This has led the WHO to list vaccine hesitancy among the top ten threats to global health in 2019.

In sharp contrast to that, in low-income countries, measles flare-ups are still rightly recognized as very serious threats to public health. This global imbalance is particularly ethically relevant, as vaccine-preventable communicable diseases may be exported from financially prosperous countries with good medical infrastructure via global migration, tourism, and trade to countries less equipped to deal with outbreaks (for instance due to problems in vaccine coverage and limited treatment options for infectious diseases). Thus, low-income countries are disproportionately affected and are far more likely to suffer serious repercussions. This situation is exacerbated by an often worse disease outcome due to prevalent malnutrition and pre-existing chronic health conditions such as tuberculosis and HIV. Consequently, neglect or refusal to be vaccinated for non-medical reasons in countries where safe and efficient measles vaccines are easily available, does not only compromise herd immunity locally, but also fails to protect more vulnerable populations globally.

This can arguably be seen in the recent measles epidemic in Samoa, where so far a total of 3149 cases have been reported, mainly young children, at least 42 of whom have died [3]. The current situation in Samoa was stirred up by a breakdown in vaccine coverage last year, with the proportion of infants receiving their second dose falling from 77% in 2017 to 28% in 2018. (ibid.) Similarly, the Democratic Republic of Congo has been heavily affected by a measles epidemic with 5110 people being declared dead since February 2019, more than twice the number of people killed by Ebola there [4].

**Figure 1:**
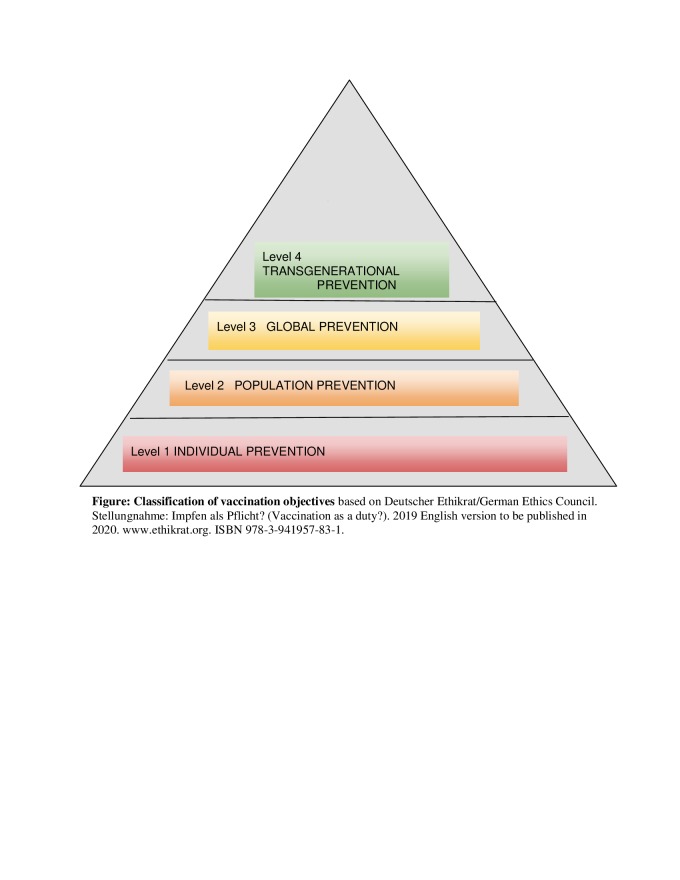
Classification of vaccination objectives.

Beyond the responsibility of each vaccinable person, the systematic and coordinated use of vaccines against exclusively human-to-human transmissible pathogens is not only capable of eliminating a particular disease locally today, but gives the current generation the opportunity to spare future generations forever from a specific source of harm through permanent global disease eradication. This context can be illustrated by the following graded classification of the individual and collective aims of vaccination, with their implication for transgenerational justice (see **Figure 1**).

Hereby, it is important to distinguish between the terms “elimination” and “eradication”. “Elimination” refers to the extinction of a particular disease in a defined geographical area with no new endemic cases of infection within that area for a defined period of time, whereas “eradication” refers to the permanent global extinction (reduction to zero of the worldwide incidence of infection) [5].

Vaccines as instruments of **individual prevention** *(Level 1)* serve the purpose of protecting a person from possible adverse short- and long-term consequences resulting from an infection with a particular pathogen. Prerequisites hereby are cheap, readily available, and safe vaccines whose benefits outweigh potential side-effects, as ascertained for the measles vaccine. In the case of some infectious diseases that are not transmitted from human to human, but through other vectors, individual protection is the principal purpose of vaccination (eg, tetanus, tick-borne encephalitis).

However, vaccines against communicable diseases serve the double function of not only protecting individuals, but also creating **herd immunity** *(Level 2)*. Hereby, vaccine non-responders, as well as individuals who cannot be vaccinated due to age restrictions or medical contraindications, are protected through the successfully immunized general population. The objective is therefore to inhibit a sustained circulation of pathogens within a community.

**Global prevention** *(Level 3)* aims at averting the export of pathogens to regions where they are not endemic. Infections imported into such regions can lead to more severe courses and outcomes of disease, mainly due to a lack of population immunity, of sufficient health care infrastructure, and sometimes belated recognition by authorities which can lead to rapidly spreading epidemics.

Successful vaccination programs concerning exclusively human-to-human transmissible infectious diseases cannot only achieve contemporary global eradication of the given disease, hence protecting the current population, but also indefinitely shelter **future generations** (*Level 4)* from harm. If a vaccine-preventable disease is successfully eradicated, coming generations are not only spared from the dangers associated with contracting that illness, but also from the financial and logistic burden to design prevention programmes, and from the potential side-effects of such interventions.

To accelerate this process, the WHO is tasked with designing coordinated international public health strategies, assigning responsibilities to the national and regional levels. In this context, the international eradication of smallpox, declared by the WHO in 1980, cannot only be perceived as a historic grand success for global health, but also as a lasting contribution to **transgenerational justice**. Wherever feasible in terms of the pathogen’s biological properties, logistics, and safety, this process should be reproduced with a strategy adapted to the given epidemiological challenges.

An evaluation of the responsibility and feasibility to mimic the smallpox example must certainly entail an individual risk-benefit assessment for each vaccine, which implies that individuals are not exposed to disproportionate risk or burden, ie, an unacceptable probability of relevant side effects or unbearable costs. Consequently, if an individual, or a society as a whole, has the opportunity to protect themselves, others and future generations from contracting a serious, potentially lethal illness, they have not only a pragmatic, but also an ethical duty to do so, thus creating transgenerational justice. This concept transfers the widely discussed political concept of sustainability into medicine, demanding deflection of possible harm from future generations through responsible action in the present. ”*Sustainability is understood as the development of the global human society toward a state of balance *(...)* between human needs and the protection of stable, functioning, life-sustaining ecological systems. *(...)* It is in its essence about transgenerational justice, ie, caring for humans living today and those living tomorrow, while preserving the integrity of the planetary ecosystem*” [6].

Evidently, the evolution and spread of new pathogens can neither be predicted nor entirely prevented, but rather managed, as seen in recent epidemics (eg, Zika, Ebola, MERS, COVID-19). Anyway, we must do our best to protect even distant future fellow human beings from a currently existing disease as soon as we have the tools to do so.

It is important to note that significant progress in vaccine compliance and consequential disease eradication can only be made through cooperation of local health care providers with international and national institutions as well as regulatory bodies. Simultaneously, every person with access to a well-functioning health care system can make a small but indispensable individual contribution to herd immunity by deciding to get vaccinated. All health care authorities are obliged to provide the means to do so, and if unable from their own resources, with access to supranational support. In this context, the implementation of vaccination policies with the aim of permanent global disease eradication, fulfilling the demands of transgenerational justice, should follow an equity-based framework of international burden sharing, with wealthy countries making proportionately higher financial and logistic contributions.

**Figure:**
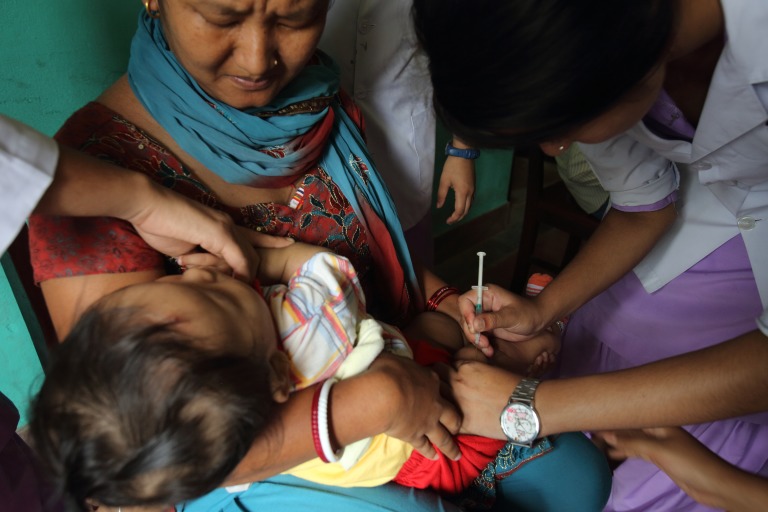
Photo: Mother brings her child to be vaccinated for Measles, Mumps and Rubella during routine vaccinations at District Public Health Office, Immunisation Clinic, Pokhara, Nepal. Photo by Jim Holmes for AusAID (13/2529).

The regulatory measures in reaction to vaccine incompliance must be region, country and population sub-group specific, customized to the given epidemiological challenges. Primarily, barriers to individual vaccine access must be removed through investment in medical infrastructure and health education, before considering penalties for non-vaccination. Whether to apply sanctions to “non-vaxxers” (due to non-medical reasons) or doctors who refuse to vaccinate, (like in some eastern European countries), or whether to reward vaccination financially (as practiced in Australia through tax benefits) or whether to exclude non-vaccinated persons, particularly children, from public institutions (as done in Italy, France, and Germany) is a decision which can only be reached taking into account the individual legal, cultural, and ethical circumstances of the given situation. However, a mandatory vaccination policy for certain occupational groups at increased risk of contracting vaccine-preventable infectious diseases certainly makes sense. Incompliance of such professionals could be sanctioned with the withdrawal of their work permit or title. This strict policy should primarily affect health care workers, childcare, and education staff, ethically justified by their freely chosen professional
